# Extracellular signal-regulated kinase 8-mediated NF-κB activation increases sensitivity of human lung cancer cells to arsenic trioxide

**DOI:** 10.18632/oncotarget.17100

**Published:** 2017-04-13

**Authors:** Dan-Dan Wu, Andy T.Y. Lau, Fei-Yuan Yu, Na-Li Cai, Li-Juan Dai, Myoung Ok Kim, Dong-Yan Jin, Yan-Ming Xu

**Affiliations:** ^1^ Laboratory of Cancer Biology and Epigenetics, Department of Cell Biology and Genetics, Shantou University Medical College, Shantou, Guangdong, P.R. China; ^2^ Department of Animal Science, Kyungpook National University, Republic of Korea; ^3^ School of Biomedical Sciences, Li Ka Shing Faculty of Medicine, The University of Hong Kong, Hong Kong, P.R. China

**Keywords:** arsenic trioxide, ERK8, IκBα, MAPK15, nuclear factor-kappaB

## Abstract

Extracellular signal-regulated kinase 8 (ERK8), also known as mitogen-activated protein kinase 15 (MAPK15), is the most recently identified protein kinase of the ERK family members and yet the least has been studied so far. Here, we report that ERK8 is highly expressed in several human lung cancer cell lines and is positively correlated with their sensitivities to the anti-cancer drug arsenic trioxide (As_2_O_3_). As_2_O_3_ at physiologically relevant concentrations (5–20 μM) potently stimulates the phosphorylation of ERK8 at Thr^175^ and Tyr^177^ within the TEY motif in the kinase domain, leading to its activation. Interestingly, activated ERK8 interacts and directly phosphorylates IkappaBalpha (IκBα) at Ser^32^ and Ser^36^, resulting in IκBα degradation. This in turn promotes nuclear factor-kappaB (NF-κB) p65 nuclear translocation and chromatin-binding, as well as the subsequent induction and activation of proteins involved in apoptosis. We also show that stable short-hairpin RNA-specific knockdown of endogenous ERK8 or inhibition of NF-κB activity by NF-κB inhibitor in high ERK8 expressing lung cancer H1299 cells blunted the As_2_O_3_-induced NF-κB activation and cytotoxicity towards these cells, indicating the critical role of ERK8 and NF-κB in mediating the As_2_O_3_ effects. Taken together, our findings suggest for the first time a regulatory paradigm of NF-κB activation by ERK8 upon As_2_O_3_ treatment in human lung cancer cells; and implicate a potential therapeutic advantage of As_2_O_3_ that might gain more selective killing of cancer cells with high ERK8 expression.

## INTRODUCTION

Human ERK8 (alias MAPK15), located at 8q24.3, is the most recently identified member of the ERK family [1]. Over the past decades, despite the fact that in-depth investigations have been done on typical ERKs (ERK1 and 2), the functions of ERK8 remain relatively understudied, in which there are still a lot of unanswered questions for its cellular function. ERK8 resembles ERK1, ERK2, and ERK5 that possesses a Thr-Glu-Tyr motif in the activation loop [1]. ERK8 has been considered as an atypical MAPK partly because it contains the long carboxyl-terminal domain that regulating its activity, cellular localization and function [1–3]. Furthermore, ERK8 is regulated by auto-phosphorylation and no specific upstream activating kinase has been identified so far [1, 4, 5]. Earlier studies from several research groups have shown that ERK8 activity can be stimulated by serum, DNA damage, and human oncogenes such as *RET/PTC3* (an active form of the *RET* proto-oncogene) [1, 6, 7]. Moreover, ERK8 has recently been shown to increase tumorigenesis in human colon cancer cells by activating c-jun and in gastric cancer cells by stabilizing c-Jun [8, 9]. Besides, ERK8 is involved in maintaining genome stability as well as autophagy [10, 11]. Yet, it is still unclear whether ERK8 acts as a proto-oncogene or tumor suppressor. However, it should be noted that protein expressed in one cell type might actually function differently in another, leading to diverse phenotypes. Therefore, no unified functions of ERK8 can be drawn conclusively at present.

Arsenic trioxide (As_2_O_3_), a traditional Chinese medicine, inhibits growth and promotes apoptosis in many different cancer cell types. It has been proven especially to be highly effective against hematologic malignancies, such as acute promyelocytic leukemia (APL) [12, 13]. Moreover, promising preclinical activity of As_2_O_3_ against malignancies other than APL was noted, such as myeloid leukemia, lymphoma, lymphocytic leukemia, and solid tumor cell lines of prostate, cervix, bladder, ovary, colon, stomach, and esophagus [12]. Lung cancers are malignant tumors with high incidences in China and worldwide [14] and characterized with high mortality because of the development of acquired resistance to chemotherapy [15]. Although recent studies have shed light on the potential of As_2_O_3_ against human lung cancers [16–21], however, there are still missing links to be explored.

In this study, we provide evidence to show that ERK8 is highly expressed in several human lung cancer cell lines. Remarkably, we report for the first time that As_2_O_3_ at physiologically relevant concentrations (effective in as low as ∼5 μM) induces the phosphorylation of ERK8 and activated ERK8 subsequently promotes the phosphorylation and degradation of IκBα, which leads to the activation of NF-κB and lung cancer cell apoptosis. The pro-apoptotic role of ERK8 and NF-κB played in As_2_O_3_ cytotoxicity has been supported by the fact that short-hairpin RNA-specific knockdown of ERK8 or inhibition of NF-κB activity by NF-κB inhibitor in high ERK8-expressing human lung cancer H1299 cells blunted the As_2_O_3_-induced NF-κB activation and cytotoxicity towards these cells, indicating that both ERK8 and NF-κB are critical players in mediating the effects of As_2_O_3_. Taken together, our findings establish a novel regulatory circuit of NF-κB activation by ERK8 upon As_2_O_3_ treatment, and implicate the potential of As_2_O_3_ in targeting lung cancers with high ERK8 expression.

## RESULTS

### As_2_O_3_ induces the phosphorylation of ERK8

**Figure 1:**
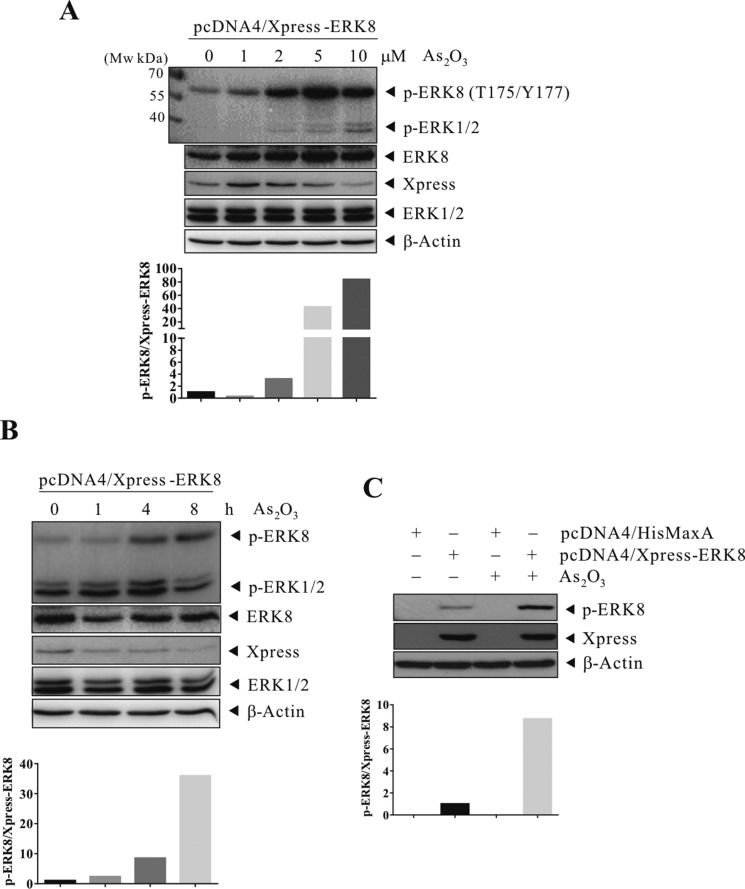
Activation of ERK8 by As_2_O_3_ in a dose and time-dependent manner in Xpress-ERK8 transfected cells HEK293T cells were transfected with pcDNA4/HisMaxA control or pcDNA4/Xpress-ERK8 expression plasmids (6 μg). At 24 h post-transfection, they were cultured in serum-free medium for 24 h, after which they were sham exposed or exposed to As_2_O_3_ and monitored. (**A**) 1 to 10 μM As_2_O_3_ for 4 h; (**B**) 5 μM As_2_O_3_ for 1 to 8 h; and (**C**) 5 μM As_2_O_3_ for 4 h. Cells were lysed, and protein extracts were subjected to SDS-PAGE followed by immunobloting using antibodies against p-ERK8 (Thr^175^/Tyr^177^). After development, the membranes were stripped and reprobed with regular antibodies against ERK8, Xpress, ERK1/2, and β-actin to monitor the total level of ERK8, ERK1/2, and loading difference, respectively. The data are representative of three independent experiments.

Our group has been investigating novel functions of oncokinases and their downstream substrates as potential signaling axis/molecular targets for cancer intervention. Here, we focused on novel functions and signaling cascade mediated by ERK8. Previously, ERK8 has been shown to be phosphorylated and activated by serum and growth factors such as epidermal growth factor (EGF) and increased tumorigenesis of human colon cancer [8]. However, other stimuli that would lead to its activation is unclear and the involvement of ERK8 in ROS stress/redox signaling is largely unexplored. Among the ROS-inducing drugs, we are particularly keen on As_2_O_3_ as it is a well-known anti-cancer therapeutic agent with promising efficacy on hematologic malignancies such as APL. To determine whether ERK8 can be activated by As_2_O_3_, we transfect Xpress-ERK8 in HEK293T cells and they were subsequently exposed to As_2_O_3_. As the phosphorylation of ERK8 at Thr^175^ and Tyr^177^ signifies its kinase activation status and therefore we detected the levels of p-ERK8 at Thr^175^ and Tyr^177^ upon As_2_O_3_ treatment in these cells by western blot analyses. Surprisingly, from the results, it can be seen that the phosphorylation of ERK8 is increased by As_2_O_3_ treatment in a dose- and time-dependent manner, suggesting that ERK8 can be activated by As_2_O_3_ and that ERK8 may mediate the cellular effects of As_2_O_3_ (Figure 1A–1C). This prompts us further to delineate the possible relationship of As_2_O_3_ and ERK8 and the downstream targets.

### IκBα as a novel substrate of ERK8

**Figure 2:**
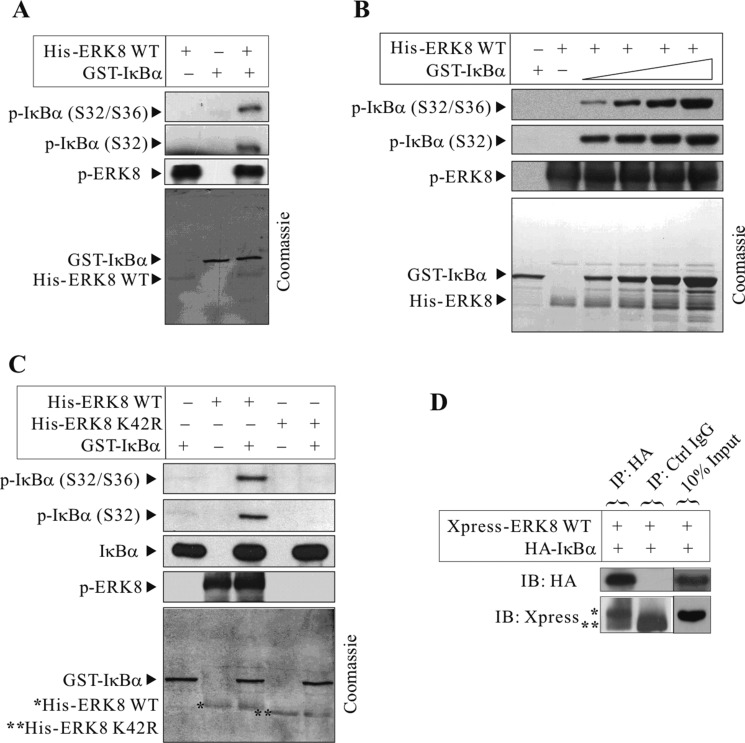
ERK8 phosphorylates and interacts with IκBα *In vitro* kinase assays were conducted using (**A**) recombinant active His-ERK8 WT and GST-IκBα; (**B**) recombinant active His-ERK8 WT and increasing amounts of GST-IκBα; and (**C**) recombinant active (WT) or kinase dead (K42R) His-ERK8 and GST-IκBα as described in “Materials and methods”. Western blot analyses of IκBα phosphorylation after the kinase assays using phospho-specific antibody against p-IκBα (Ser^32^) or p-IκBα (Ser^32^/Ser^36^) were conducted. p-ERK8 levels signifies its kinase activity which could be detected in active His-ERK8 WT but not the ERK8 K42R mutant. (**D**) Xpress-ERK8 WT and HA-IκBα expression plasmids (3 μg of each) were transiently transfected into HEK293T cells. At 24 h post-transfection, they were cultured in serum-free medium for 24 h, after which they were exposed to 10 μM MG132 for 4 h. IκBα was immunoprecipitated (IP) with anti-HA or Ctrl IgG agarose beads followed by immunoblotting with anti-HA or anti-Xpress antibodies (*, Xpress-ERK8 WT; **, IgG heavy chain). For (A), (B), and (C), the corresponding gels are stained with Coomassie Blue to monitor equal protein loading (bottom panels of each). In (C), *, His-ERK8 WT; **, His-ERK8 K42R). The data are representative of three independent experiments.

To date, knowledge about the cellular physiological protein substrate(s) of ERK8 is limited. Previous studies have shown that c-Jun is an ERK8 substrate and related to colon and gastric cancer development [8, 9]. For this reason, we sought to discover novel ERK8 substrates and we are particular keen on redox signaling pathways since we found that ERK8 can be activated by the oxidant As_2_O_3_. To identify novel substrates of ERK8, we first expressed and purified active His-tagged ERK8 and perform *in vitro* kinase assay using a list of potential substrates available in our laboratory. Remarkably, among these substrates, we found that IκBα can be robustly phosphorylated by ERK8 at Ser^32^ and Ser^36^ as determined by western blot analyses (Figure 2A and 2B), but not the ERK8 K42R kinase-dead mutant (Figure 2C).

To determine whether ERK8 and IκBα interact each other, plasmids encoding HA-IκBα and Xpress-ERK8 were cotransfected into HEK293T cells. IκBα proteins were immunoprecipitated with anti-HA agarose beads and subjected to western blot analyses using anti-Xpress antibody. ERK8 was confirmed to interact with IκBα *ex vivo* (Figure 2D). From the results, we reported for the first time that IκBα is a novel substrate of ERK8 and the likelihood of ERK8 in regulating NF-κB pathway.

### ERK8 promotes the phosphorylation and degradation of IκBα

**Figure 3:**
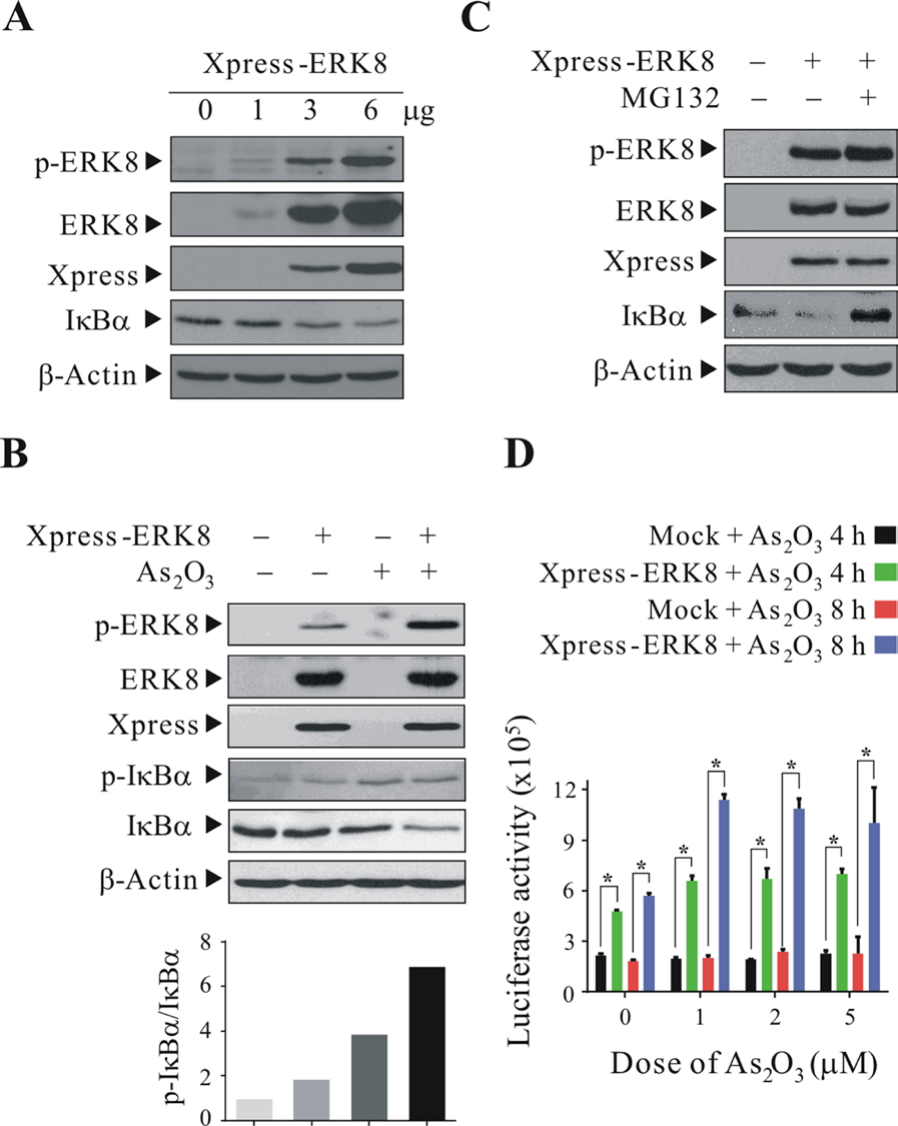
ERK8 promotes the phosphorylation and degradation of IκBα HEK293T cells were transfected with Xpress-ERK8 expression plasmid. At 24 h post-transfection, they were cultured in serum-free medium for 24 h, after which they were exposed to 5 μM As_2_O_3_ for 4 h. (**A**) cells transfected with 1 to 6 μg Xpress-ERK8 expression plasmid; (**B**) cells transfected with 6 μg Xpress-ERK8 expression plasmid; (**C**) cells transfected with 6 μg Xpress-ERK8 expression plasmid, and prior to As_2_O_3_ treatment, cells were first pretreated with 10 μM MG132 for 4 h. Cells were lysed, and protein extracts were subjected to SDS-PAGE followed by immunobloting using antibodies against p-ERK8 and p-IκBα (Ser^32^/Ser^36^). After development, the membranes were stripped and reprobed with regular antibodies against ERK8, Xpress, IκBα, and β-actin to monitor the total level of ERK8, IκBα and loading difference, respectively. The intensities of the bands of p-IκBα and IκBα in (B) were quantified and expressed as relative ratios, setting 1 for control. (**D**) NF-κB luciferase assay using NIH/3T3 cells treated with As_2_O_3_. NIH/3T3 cells seeded at 6 cm dish were transient transfected with ERK8 expressing plasmid (6 μg) along with the 5× kB luciferase reporter plasmid (6 μg) and the *Renilla* luciferase reporter plasmid (150 ng). At 24 h post-transfection, they were seeded into 24 well-plate at 1 × 10^5^ cells/well. After 24 h, they were sham-exposed or exposed to 1, 2, or 5 μM As_2_O_3_ for additional 4 and 8 h. NF-κB-luciferase activity was measured and normalized against *Renilla* luciferase activity as described in “Materials and methods”. *A significant difference of *P* < 0.05. The data are representative of three independent experiments.

Among the redox sensitive transcription factors, NF-κB is regulated by its inhibitor IκBα which sequesters NF-κB from entering the nucleus. Upon stimulus, phosphorylation of IκBα at Ser^32^ and Ser^36^ would lead to the dissociation of IκBα from NF-κB and its subsequent ubiquitin-proteasomal degradation; as a result, NF-κB is translocated from the cytosol into nucleus and activates NF-κB-dependent gene expression [22]. To investigate the cellular physiological role of ERK8 in modulation of NF-κB pathway, first, we transfected Xpress-ERK8 into HEK293T cells and they were subsequently treated with 5 μM As_2_O_3_ for 4 h. From the western blot results, we observed that ectopically-expressed ERK8 can be activated by As_2_O_3_ treatment and the p-ERK8 levels positively correlate to the amount of the ERK8 plasmid transfected (Figure 3A). Moreover, if IκBα is a physiological substrate of ERK8 and ERK8 would catalyze the phosphorylation of IκBα at Ser^32^/Ser^36^ in cells, then ERK8 should also presumably promote the degradation of IκBα. As expected, when we transfect an increasing amount (1 to 6 μg) of Xpress-ERK8 expression plasmid into cells and subsequently dosed them with 5 μM As_2_O_3_, the expression level of endogenous IκBα declined accordingly (Figure 3A). To correlate if the decline of IκBα level is due to phosphorylation, we perform similar experiments and also monitor the level of p-IκBα at Ser^32^/Ser^36^. From the results, it can be seen that there is an obvious enhancement of phosphorylation of IκBα at Ser^32^/Ser^36^ in ERK8 transfected cells with As_2_O_3_ treatment (Figure 3B, lane 4). Although there is also elevation of the p-IκBα level in As_2_O_3_-treated cells (Figure 3B, lane 3), however, the protein level of IκBα declined more dramatically in Xpress-ERK8 transfected cells with As_2_O_3_ treatment (Figure 3B, lane 4). The ratio of p-IκBα/IκBα has the highest value in Xpress-ERK8 transfected cells with As_2_O_3_ treatment, reflecting that endogenous IκBα protein level is rapidly declined. To further substantiate the notion that the decline of IκBα protein level is due to its proteasomal degradation, we pretreated these cells with the proteasome inhibitor MG132 before exposure to As_2_O_3_. From the results, treatment of MG132 prior to As_2_O_3_ treatment prevented the decline of IκBα upon As_2_O_3_ treatment (compare lane 2 to lane 3 in Figure 3C). To determine whether IκBα degradation would lead to enhanced NF-κB activity, we performed NF-κB luciferase assay by cotransfecting the 5× κB firefly luciferase reporter with Xpress-ERK8 expression plasmid in NIH/3T3 cells and then challenged the cells with 5 μM As_2_O_3_ for 4 h. Our results indicate that As_2_O_3_ can synergize ERK8 in promotion of NF-κB activity (Figure 3D).

### ERK8 promotes the phosphorylation and degradation of IκBα, as well as nuclear translocation of NF-κB in human lung cancer cells

**Figure 4:**
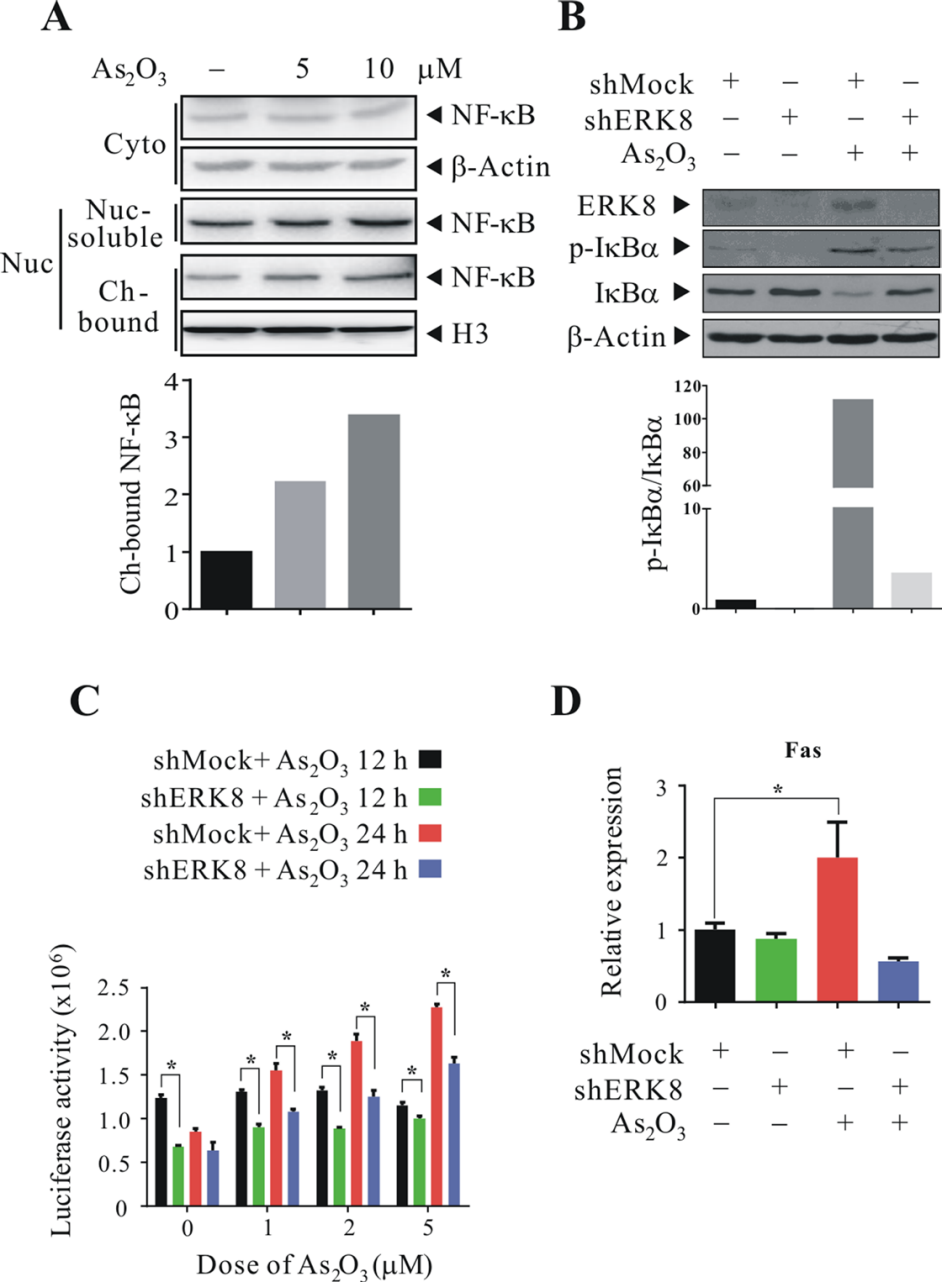
ERK8 promotes the phosphorylation and degradation of IκBα, as well as nuclear translocation of NF-κB in human lung cancer cells (**A**) H1299 cells were treated with 5 μM As_2_O_3_ for 4 h; individual subcellular fractions (cytoplasmic, nuclear-soluble, and chromatin-bound) were isolated and subjected to western blot analysis using NF-κB p65 antibody. The same blot was stripped and reprobed with the monoclonal β-actin or histone H3 antibody to monitor the loading difference. The intensities of the bands of chromatin-bound NF-κB and histone H3 were quantified and expressed as relative ratios, setting 1 for control. (**B**) Stable mock control (shMock) and ERK8 knockdown (shERK8) H1299 cells were cultured in serum-free medium for 24 h, after which they were exposed to 5 μM As_2_O_3_ for 4 h. Cells were lysed, and protein extracts were subjected to SDS-PAGE followed by immunobloting using antibodies against ERK8 and p-IκBα (Ser^32^/Ser^36^). After development, the membranes were stripped and reprobed with regular antibodies against IκBα and β-actin to monitor the total level of IκBα and loading difference, respectively. The intensities of the bands of p-IκBα and IκBα were quantified and expressed as relative ratios, setting 1 for control. (**C**) NF-κB luciferase assay using shMock and shERK8 H1299 cells treated with As_2_O_3_. Stable shMock and shERK8 H1299 cells at 6 cm dish were transient transfected with the 5× κB luciferase reporter plasmid (6 μg) and the *Renilla* luciferase reporter plasmid (150 ng). At 24 h post-transfection, they were seeded into 24 well-plate at 1 × 10^5^ cells/well. After 24 h, they were sham-exposed or exposed to 1, 2, or 5 μM As_2_O_3_ for additional 12 and 24 h. NF-κB-luciferase activity was measured and normalized against *Renilla* luciferase activity as described in “Materials and methods”. (**D**) Quantitative real-time RT-PCR to determine the mRNA level of NF-κB downstream gene Fas in stable shMock and shERK8 H1299 cells treated with 5 μM As_2_O_3_ after serum starvation for 24 h. β-Actin was used as an internal control. *A significant difference of *P* < 0.05. The data are representative of three independent experiments.

The results thus far suggested a potential paradigm of NF-κB activation by ERK8. However, whether similar phenomenon occurs in other cells would need to be verified. As our team has been focusing on lung-related disease, especially lung cancers; for this reason, we screened the basal levels of ERK8 in several human lung cancer cell lines available in our laboratory, including Calu-3, H1299, H358, and H460, by quantitative real-time RT-PCR and western blot analyses. From the results, it can be seen that ERK8 is expressed at low level in Calu-3 cells; however, among the lung cancer cells, ERK8 has a relatively high expression in H1299, H358, and H460 cells (Supplementary Figure 1A and 1B). To determine whether there is NF-κB activation in high ERK8 expressing cells, we checked on H1299 cells since the subcellular fractionation quality turns out to be the best among these cell lines. So, after we treated H1299 cells with 5 μM As_2_O_3_, we fractionated individual subcellular components and it was found that the levels of nuclear NF-κB in nuclear-soluble and chromatin-bound fractions increased as compared with untreated control (Figure 4A). Similar results were observed in H358 cells (data not shown). This indicates that there is an enhanced NF-κB chromatin-binding in As_2_O_3_-treated cells, and suggests that NF-κB might likely be involved in modulating the subsequent gene expressions. To further strengthen the relationship of As_2_O_3_-ERK8-IκBα-NF-κB cascade, we generate H1299 stable ERK8 knockdown cells and examine the levels of p-IκBα and IκBα upon As_2_O_3_ treatment. From our results, knocking down ERK8 in H1299 cells blunted the phosphorylation and degradation of IκBα (compare lane 3 to lane 4 in Figure 4B), and as a result sustained the ratio of p-IκBα/IκBα close to untreated controls. Furthermore, we also performed NF-κB luciferase assay by transfecting the 5× κB firefly luciferase reporter in these shMock and shERK8 cells and then challenged the cells with 5 μM As_2_O_3_. Our results indicate that the NF-κB luciferase activity is significantly suppressed in ERK8 knockdown cells as compared to shMock control (Figure 4C). In addition, the mRNA level of NF-κB downstream genes Fas [23, 24] was detected to further confirm the activation of NF-κB. Interestingly, the expression of Fas increased in shMock but not in stable shERK8 H1299 cells upon exposure to As_2_O_3_ (Figure 4D). These results suggest that ERK8 is critical in mediating the effect of As_2_O_3_ and the subsequent NF-κB activation in these cells.

### As_2_O_3_ promotes cell death in high ERK8-expressing lung cancers

**Figure 5:**
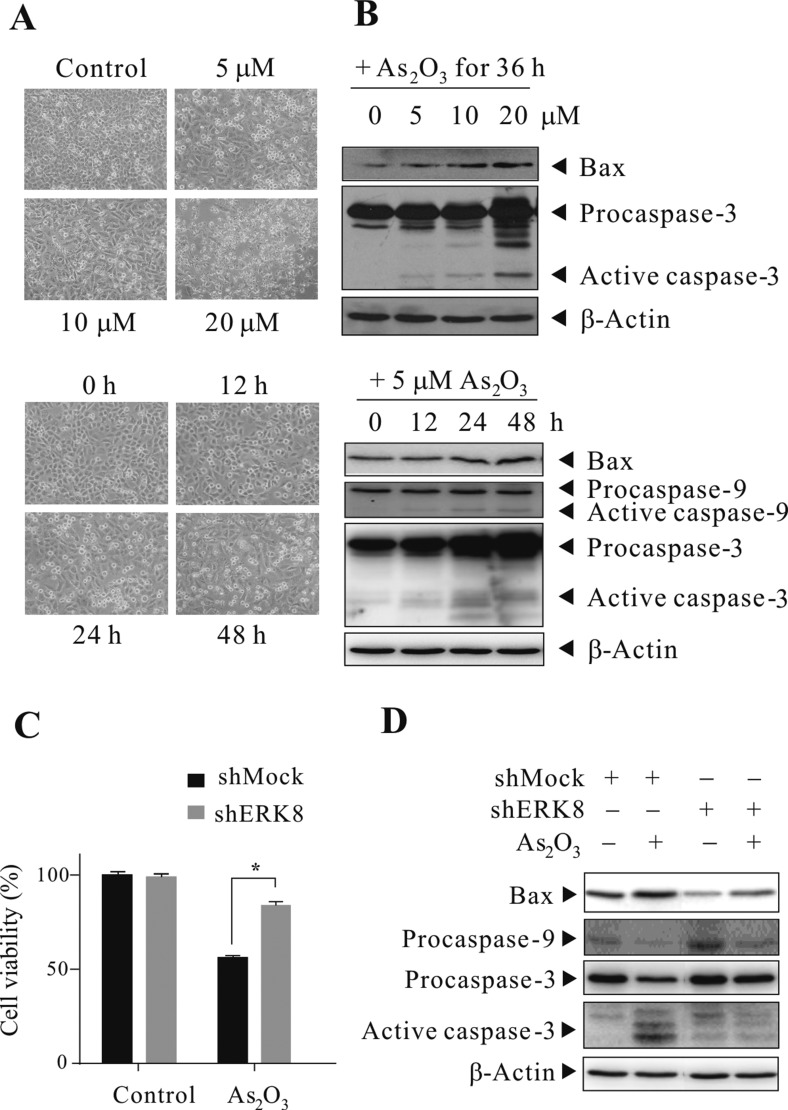
As_2_O_3_ promotes cell death in high ERK8-expressing lung cancers Dose- and time-dependent cytotoxicity of As_2_O_3_ towards H1299 cells. H1299 cells were sham-exposed or exposed to 5-20 μM of As_2_O_3_ for 36 h or 5 μM As_2_O_3_ for 12-48 h. (**A**) cell morphology of H1299 cells under light microscope. (**B**) The corresponding western blot of (A), cells were lysed and protein extracts were subjected to western blot analyses using antibodies against Bax, Caspase-9, and Caspase-3. After development, the membranes were stripped and reprobed with β-actin to monitor the loading difference. (**C**) Stable shMock and shERK8 H1299 cells were sham-exposed or exposed to 5 μM As_2_O_3_ for 48 h, the percentage of cell viability was determined by MTS assay. *A significant difference of *P* < 0.05. (**D**) The corresponding western blot of (C) for the determination of marker proteins involved in apoptosis. The data are representative of three independent experiments.

From the above sections, we observed the activation of ERK8 by As_2_O_3_ treatment, with the concomitant phosphorylation and degradation of IκBα, as well as induction of NF-κB activity, but whether this would ultimately impact on cell growth or cell death regulation is unclear. For this reason, we sought to study the phenotype of cells mediated by this regulatory circuit. As mentioned earlier, since there is a relatively high ERK8 expression in H1299 cells, we would therefore want to know the outcome of As_2_O_3_ treatment to this high ERK8-expressing lung cancer cell line. H1299 cells were sham-exposed or treated with As_2_O_3_ (5 to 20 μM) and monitored, it can be seen that massive cytotoxicity occurred in a dose- and time-dependent manner in this cell line (Figure 5A). To check whether the observed cytotoxicity is due to the execution of apoptosis, we checked the expression levels of several important apoptosis mediators by western blot analysis. The induction/cleavage of BAX/CASP9/CASP3 can well be observed (Figure 5B), suggesting that As_2_O_3_ treatment indeed leads to apoptotic cell death. As a proof of concept, we perform similar experiments on shMock and shERK8 H1299 cells. This time, the cell viabilities were monitored by MTS assay post 48 h of As_2_O_3_ treatment. Notably, knocking down ERK8 in H1299 cells sustained the cell viability of shERK8 cells upon As_2_O_3_ treatment, but not in shMock cells (Figure 5C). Similar results were observed in H460 cells (data not shown). In addition, knocking down ERK8 in H1299 cells blunted the induction/cleavage of marker proteins involved in apoptosis (Figure 5D).

### NF-κB specific inhibitor, JSH-23, inhibits ERK8 promoted As_2_O_3_-induced lung cancer cell death

**Figure 6:**
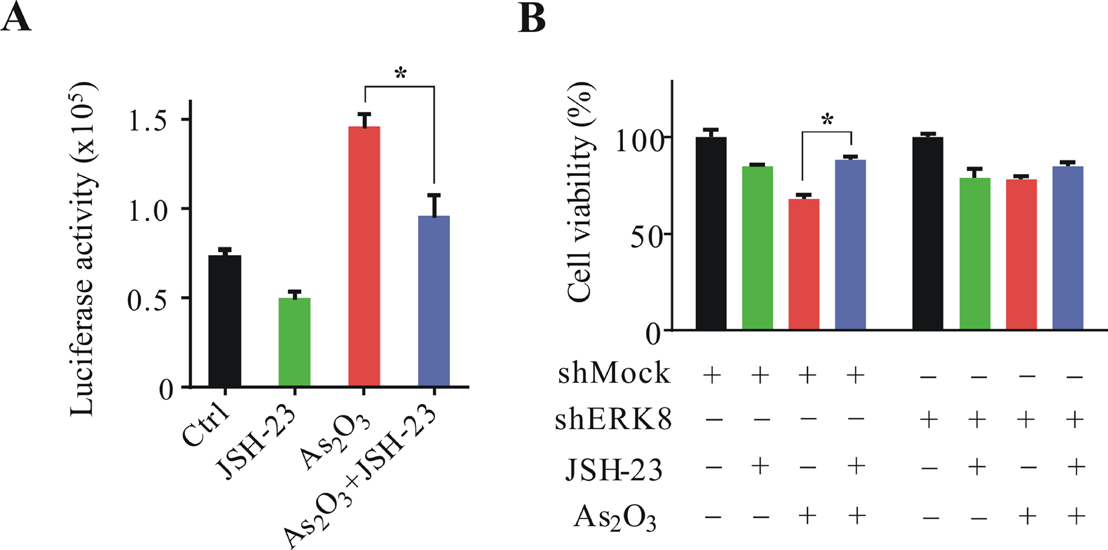
JSH-23 inhibits ERK8 promoted As_2_O_3_-induced lung cancer cell death (**A**) NF-κB luciferase assay of H1299 cells in the presence or absence of JSH-23 (30 μM) post 12 h exposure to As_2_O_3_ (5 μM). The NF-κB luciferase activity was determined similarly as described in the previous sections. (**B**) Stable shMock and shERK8 H1299 cells were exposed to As_2_O_3_ (5 μM) in the presence or absence of JSH-23 (30 μM) for 36 h, the cell viability was determined by NBB assay. All the results were expressed as the mean ± SD of triplicate samples and the reproducibility was confirmed in three separate experiments. *A significant difference of *P* < 0.05.

To further confirm that ERK8 promotes As_2_O_3_-induced lung cancer cell apoptosis mainly and specifically through IκBα associated NF-κB signaling, the NF-κB specific inhibitor (JSH-23) was employed. First, NF-κB luciferase assay was performed to detect the effectiveness of JSH-23 on the suppression of NF-κB activity. As we can see in Figure 6A, JSH-23 treatment significantly suppressed As_2_O_3_-induced NF-κB luciferase activity in H1299 cells. To correlate the sensitivity of H1299 cells to As_2_O_3_ is due to ERK8 mediated NF-κB activation, we performed cell viability assay in stable shMock and shERK8 H1299 cells. As expected, JSH-23 treatment significantly sustained the cell viability of As_2_O_3_-treated shMock H1299 cells (compare column 3 to column 4 in Figure 6B). In the previous section, since we showed that knocking down ERK8 significantly suppressed the NF-κB activity and sustained the cell viability of shERK8 H1299 cells upon As_2_O_3_ treatment (Figures 4C and 5C), therefore, no significant difference in As_2_O_3_ sensitivity was observed at all between JSH-23-untreated or JSH-23-treated shERK8 H1299 cells (compare column 7 to column 8 in Figure 6B). These data indicate that lowering the ERK8 level alone is sufficient and reminiscent to reducing the activity of NF-κB signaling, which ultimately diminished the cellular sensitivity to As_2_O_3_. Altogether, our data suggest that As_2_O_3_ is very effective in promoting cell death regulation in high ERK8-expressing lung cancer cells and we document for the first time on the interplay of As_2_O_3_-ERK8-IκBα-NF-κB signaling axis.

## DISCUSSION

Our group has been investigating novel functions of protein post-translational modifications. In the present study, we focused on novel substrates of ERK8. Previously, it has been reported that ERK8 is overexpressed in human colon cancers [8], suggesting that ERK8 might be a promising chemotherapeutic target. Here, we first wonder whether ERK8 may be overexpressed as well in lung cancers. For this reason, we screened the levels of ERK8 in several human lung cancer cell lines available in our lab, including Calu-3, H1299, H358, and H460, by quantitative real-time RT-PCR and western blot analyses. From the results, it can be seen that ERK8 is expressed at low level in Calu-3 cells. However, among the lung cancer cells, ERK8 has a relatively high expression in H1299, H358, and H460 cells (Supplementary Figure 1A and 1B).

**Figure 7:**
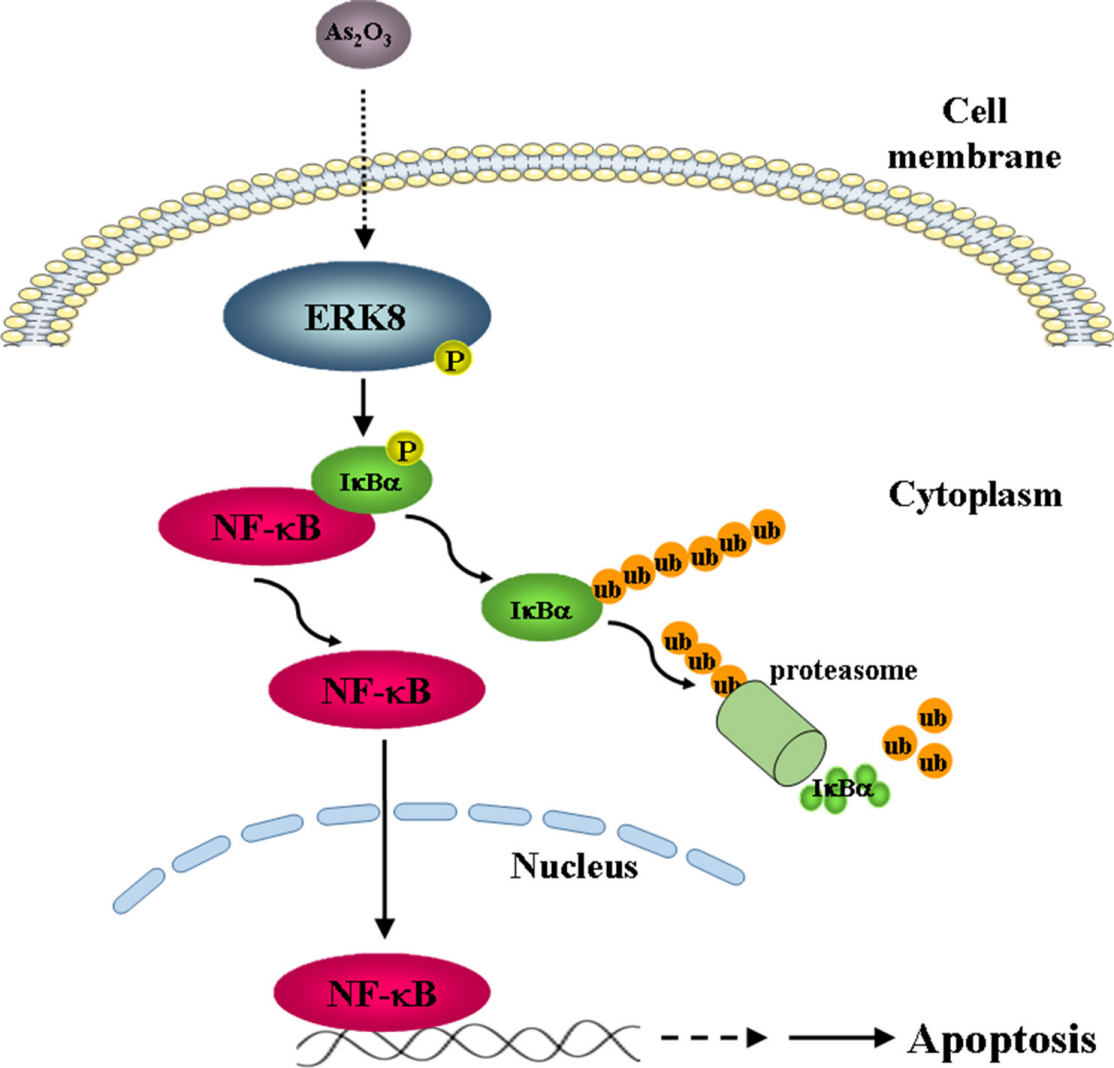
A model of As_2_O_3_ action based on our data is presented As_2_O_3_ treatment induces the phosphorylation of ERK8 and activated ERK8 subsequently promotes the phosphorylation and degradation of IκBα, which leads to the activation of NF-κB and lung cancer cell apoptosis.

The traditional Chinese medicine As_2_O_3_ is a well-known anti-cancer therapeutic agent with promising efficacy on hematologic malignancies such as APL. Interestingly, we found that As_2_O_3_ induced preferential cytotoxicity in several high ERK8-expressing lung cancer cells (H1299, H358, and H460) (Figure 5; Supplementary Figure 1C). Indeed, the pivotal role of ERK8 that it plays in mediating the effect of As_2_O_3_ has been demonstrated in our study, in which shRNA knockdown of ERK8 or inhibition of NF-κB activity by JSH-23 treatment blunted the As_2_O_3_-induced NF-κB activation and cell death in H1299 cells. Although the phosphorylation of ERK1/2 and p38 MAPK also increased slightly by As_2_O_3_ treatment (Figure 1A and 1B; Supplementary Figure 2), however, the magnitude of ERK8 phosphorylation is significantly higher as compared to those of ERK1/2 and p38 MAPK in all the respective time points and As_2_O_3_ dose examined, suggesting that ERK8 is dominantly activated by As_2_O_3_ in high ERK8 expressing cells (Figure 1A–1C; Supplementary Figure 2). Altogether, these data confirm that the interplay of ERK8-IκBα-NF-κB is critical for mediating the apoptotic signal exerted by As_2_O_3_ (Figure 7).

To date, besides IkappaB kinases and ribosomal S6 kinase 2 [25, 26], other potential kinases on the phosphorylation of IκBα at Ser^32^ and Ser^36^ is unknown/understudied. Moreover, knowledge about cellular physiological protein substrate(s) of ERK8 is limited. Previous studies have shown that c-Jun is an ERK8 substrate and related to colon and gastric cancer development [8, 9]. In here, we show that As_2_O_3_ can specifically promote the activation of ERK8 and the subsequent phosphorylation and degradation of IκBα, leading to NF-κB activation and inducing cell death in high ERK8 expressing human lung cancer cells. The reason of the Janus-faced role of ERK8 in promotion of cell growth and cell death may be very complex and cell type-dependent. Therefore, similar to JNK/c-Jun axis, depending on the types and magnitude of stimuli, which can either promote cell death or cell transformation; suggesting that although EGF (mitogenic stimulus) or As_2_O_3_ (apoptotic stimulus) can both activate the phosphorylation of ERK8 at Thr^175^/Tyr^177^, however, the relaying downstream targets are crucial in dictating the cell fate that ultimately lead to cell transformation or cell death. Nevertheless, further study is required to address these issues to find out the NF-κB dependent genes in response to As_2_O_3_ treatment (besides Fas as we have demonstrated here) that lead to cell death in high ERK8-expressing human lung cancer cells.

In summary, we demonstrate for the first time a regulatory paradigm of NF-κB activation by ERK8 upon As_2_O_3_ treatment and that IκBα is a newly identified cellular physiological substrate of ERK8. Given the important role of ERK8 in mediating the therapeutic efficacy of As_2_O_3_ against lung cancer cells, we suggest that As_2_O_3_ (besides its promising role in the treatment of hematologic malignancies) as a potential agent in the treatment of lung cancer patients with high ERK8 expression such that this might gain more selective killing of cancer cells. Finally, the work we report here might also implicate the need for ongoing research on the identification and understanding of novel signaling circuit(s) in response to As_2_O_3_ treatment in various cancer types, and hopefully shed light in providing strategies for anti-cancer management.

## MATERIALS AND METHODS

### Materials

Arsenic trioxide (As_2_O_3_) and MG132 were purchased from Sigma-Aldrich (St. Louis, MO). The NF-κB specific inhibitor JSH-23 (S7351) was purchased from Selleck (Shanghai, China). The Subcellular Protein Fractionation Kit for Cultured Cells was from Thermo Scientific (Rockford, IL). shRNAs were purchased from Santa Cruz Biotechnology (Santa Cruz, CA). ERK8 shRNA plasmid (h) (sc-77462-SH) is a target-specific lentiviral vector plasmid encoding a 19–25 nt (plus hairpin) shRNA to knock down gene expression. Control shRNA plasmid-A (sc-108060) encodes a scrambled shRNA sequence that will not lead to the specific degradation of any known cellular mRNA. All other general chemicals were purchased from GE Healthcare (Uppsala, Sweden), Qiagen (Valencia, CA) and Sigma-Aldrich. The pCMV4-3 HA/IκBα (21985) and pRL-SV40P (27163) plasmids were purchased from Addgene (Cambridge, MA). The 5× κB luciferase reporter plasmid has been described previously [27]. The *Homo sapiens* ERK8 gene coding sequence (NM_139021) expression plasmid pGEX-2T-GST-ERK8 wild-type (WT) and the corresponding kinase-dead mutant (K42R) were generously provided by Dr. Mark K. Abe from the Univesity of Chicago (Chicago, IL). Antibodies used for western blot were purchased from BBI Life Sciences (Shanghai, China), Santa Cruz Biotechnology, GeneTex (Irvine, CA), Cell Signaling Technology (Danvers, MA), Invitrogen (Waltham, MA) and Sigma-Aldrich, with the following dilutions: ERK8 (AB21813b; BBI Life Sciences), 1:1000; p-ERK1/2 Thr^202^/Tyr^204^ (4370; Cell Signaling Biotechnology, also being utilized for the detection of phosphorylated ERK8 at Thr^175^/Tyr^177^ as described previously), 1:1000 [8]; ERK1/2 (sc-292838; Santa Cruz Biotechnology), 1:1000; p-p38 MAPK (4511; Cell Signaling Technology), 1:1000; p38 MAPK (8690; Cell Signaling Technology), 1:1000; p-JNK (sc-293138; Santa Cruz Biotechnology), 1:1000; JNK (sc-7345; Santa Cruz Biotechnology), 1:1000; p-IκBα Ser^32^ (2859; Cell Signaling Biotechnology), 1:1000; p-IκBα Ser^32^/Ser^36^ (9246; Cell Signaling Biotechnology), 1:1000; IκBα (AB20138a; BBI Life Sciences), 1:1000; NF-κB p65 (sc-8008; Santa Cruz Biotechnology), 1:1000; Bax (GTX109683; GeneTex), 1:1000; Caspase-9 (GTX112888; GeneTex), 1:1000; Caspase-3 (GTX110543; GeneTex), 1:1000; HA-probe (sc-7392; Santa Cruz Biotechnology), 1:1000; Xpress (46-0528; Invitrogen), 1:5000; and β-Actin (A5441; Sigma-Aldrich), 1:10000.

### Construction of ERK8 expression plasmids and the corresponding mutants

The ERK8 WT and ERK8 K42R were each inserted into the pET46 Ek/LIC plasmid and expressed as His-tagged fusions as described previously [8]. The ERK8 proteins were purified using Ni-NTA agarose (Qiagen). The ERK8 gene coding sequence was also amplified from pGEX-2T-GST-ERK8 WT by PCR. After restriction digestion, the ERK8 sequence was ligated to the BamHI/XbaI site of pcDNA4/HisMaxA to generate the pcDNA4/Xpress-ERK8. For preparation of GST-IκBα, the IκBα gene coding sequence was amplified from pCMV4-3 HA/IκB-alpha by PCR. After restriction digestion, the IκB-alpha sequence was ligated to the EcoRI/NotI site of pGEX-5X-1 to generate the pGEX-5X-1/GST- IκBα. The recombinant plasmids were confirmed by DNA sequencing (Beijing Genomics Institute, Shenzhen, Guangdong, China).

### Cell culture and transfection

All the cell lines employed in this study, including human lung cancer cells (Calu-3, H1299, H358, and H460) as well as HEK293T, NIH/3T3, HL-60, HepG2, and HeLa cells, were purchased from and authenticated by the ATCC Cell Bank of the Chinese Academy of Sciences (Shanghai, China) and cultured following the supplier's recommended conditions. Cell viability was measured by MTS or NBB assay as described previously [8, 28]. Transfection of the expression vectors was performed using Lipofectamine (Invitrogen) following the manufacturer's suggested protocol. For generation of ERK8 knockdown cells, viral particles were packed with control or ERK8 shRNA. H1299 cells, grown to about 70% confluence, were infected with the above lentiviral shRNAs in the presence of 8 μg/ml polybrene for 24 h. Uninfected cells were eliminated by exposure to 2 μg/ml puromycin for 4 days before use.

### Quantitative real-time RT-PCR

In brief, total RNA was extracted from cells using TRIzol Reagent (Thermo Fisher Scientific, 15596018). cDNA was synthesized using PrimeScript Reverse Transcriptase (Takara, 2680A) according to the manufacturer's instructions. Quantitative real-time RT-PCR was performed with GoTaq qPCR Master Mix (A6001) from Promega (Fitchburg, WI) and gene-specific primers on Applied Biosystems 7500 Real-Time PCR System. β-Actin was amplified as internal reference to normalize gene expression. Relative expression of genes was calculated using 2 ΔΔCT method as described previously [29]. Primers were synthesized by Beijing Genomics Institute with the following sequences: ERK8 (forward: 5′-GACCAGAAGCCGTCCAATGT-3′, reverse: 5′-GTATCGGTGCGAAGAGAGCA-3′); Fas (forward: 5′-TGAAGGACATGGCTTAGAAGTG-3′, reverse: 5′-GG TGCAAGGGTCACAGTGTT-3′) and β-actin (forward: 5′-ATGGGTCAGAAGGATTCCTATGTG-3′, reverse: 5′-CTTCATGAGGTAGTCAGTCAGGTC-3′).

### Cell lysate preparation and conditions of western blot and immunoprecipitation

After treatment, cells were then washed thrice with ice-cold PBS, scraped into centrifuge tube, and then harvested by centrifugation at 1000× *g* for 5 min at 4°C. For subcellular protein preparation, the cytoplasmic, nuclear-soluble, and chromatin-bound fractions were prepared using the Subcellular Protein Fractionation Kit for Cultured Cells in accordance with the manufacturer. For western blot analysis, cell pellets were lysed in radioimmunoprecipitation assay buffer according to the protocol as described previously [28]. Equal amounts of proteins were fractionated on a SDS-polyacrylamide gel and transferred onto polyvinylidene difluoride membranes. The membranes were blocked with 5% nonfat dry milk in PBS containing 0.05% Tween 20 and probed with various primary antibodies. After incubation with secondary antibodies, immunoblots were visualized with the enhanced chemiluminescence detection kit (GE Healthcare). The normalized target band signal intensities of one data set among the three independent repeats were expressed as relative ratios and presented as bar graphs. For immunoprecipitation, cells were harvested at 80 to 90% confluence and proteins were extracted in NP40 cell lysis buffer [50 mmol/L Tris-HCl (pH 8.0), 150 mmol/L NaCl, 0.5% NP40] with freezing and thawing. Equal amounts of proteins were subjected to immunoprecipitation followed by western blot analysis.

### *In vitro* kinase assay

To detect IκBα phosphorylation, GST-IκBα proteins (1 μg) were mixed with purified recombinant active or kinase dead His-ERK8, 0.2 μM ATP, and 1× kinase buffer and incubated at 30°C for 30 min. The reaction was stopped by adding 5× SDS sample buffer. The samples were boiled and then separated by SDS-PAGE and visualized by western blot analyses, or Coomassie Blue staining.

### Reporter gene assay

The 5× κB luciferase reporter plasmid construct which contains the 5× NF-κB binding site was used. NF-κB activity was analyzed by transfection of the 5× κB luciferase reporter plasmid into ERK8-overexpressing or -knockdown cells. Cells were disrupted with lysis buffer (dual-luciferase reporter assay system, Promega) at room temperature for 30 min by gentle shaking, and then firefly luciferase activity was measured. The NF-κB-luciferase activity was normalized against *Renilla* luciferase activity (pRL-SV40P).

### Statistical analysis

Statistical analysis was done by using two-tailed Student's *t*-test. A *P* value of < 0.05 was considered significant. Except for western blot analyses, all other quantitative results were expressed as the mean ± SD of triplicate samples. The reproducibility of experimental results was confirmed in three separate experiments and representative data set was shown.

## SUPPLEMENTARY FIGURES


