# Supplementary material for: Extracellular signal-regulated kinase 8-mediated NF-κB activation increases sensitivity of human lung cancer cells to arsenic trioxide
Source: Oncotarget. 2017 Apr 13;8(30):49144–55. doi: 10.18632/oncotarget.17100 (PMC5564756; doi:10.18632/oncotarget.17100)
Supplement: Supplementary file 1 [file oncotarget-08-49144-s001.pdf]

Extracellular signal-regulated kinase 8-mediated NF-κB activation increases sensitivity of human lung cancer cells to arsenic trioxide

Supplementary Materials

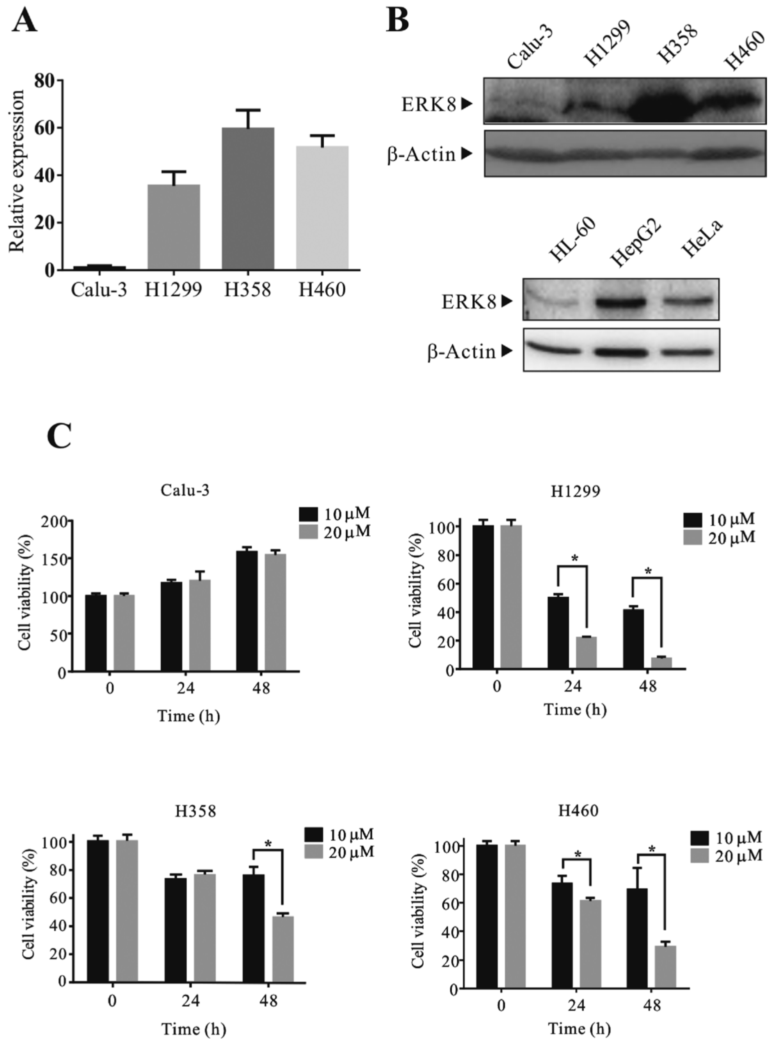

Supplementary Figure 1: Relationship of ERK8 expression levels in human lung cancer cell lines and their degree of sensitivities to the anti-cancer drug As_2O_3. (**A**) Quantitative real-time PCR for the determination of the mRNA level of ERK8 in four human lung cancer cell lines. It is a representative of three experiments. The signals were quantified and normalized with those of β-actin and plotted as a relative ratio, setting 1 for Calu-3. Results are expressed as mean ± SD of triplicate samples and reproducibility was confirmed in three separate experiments. (**B**) The corresponding western blot of (A) for the detection of the protein level of ERK8, including also cell lines from other tissues. Whole cell lysate were extracted by 1× sample buffer, antibodies against ERK8 and β-actin were used to measure the level of ERK8 and loading difference. (**C**) After 24 or 48 h of As_2O_3 treatment, the percentage of cell viability in these human lung cancer cells was determined by MTS assay. *A significant difference of $P < 0.05$. The data are representative of three independent experiments.

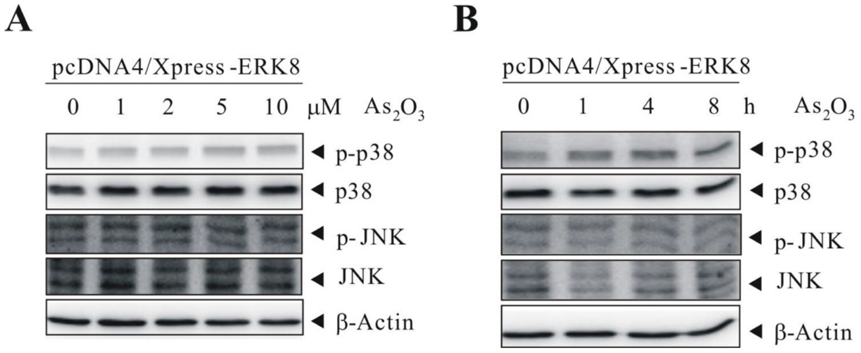

Supplementary Figure 2: The effect of As_2O_3 on JNK and p38 MAPK pathways in the presence of ERK8. HEK293T cells were transfected with pcDNA4/Xpress-ERK8 expression plasmid (6 μg). At 24 h post-transfection, they were cultured in serum-free medium for 24 h, after which they were sham exposed or exposed to As_2O_3 and monitored. (**A**) 1 to 10 μM As_2O_3 for 4 h; (**B**) 5 μM As_2O_3 for 1 to 8 h. Cells were lysed, and protein extracts were subjected to SDS-PAGE followed by immunobloting using antibodies against p-JNK, p-p38 MAPK, JNK, p38 MAPK, and β-actin to monitor the phosphorylation and total level of JNK, p38 MAPK, and loading difference, respectively. The data are representative of three independent experiments.
